# Seasonal shifts in the habitat selection patterns of male American Marten (*Martes americana*) at a fine spatial scale

**DOI:** 10.1093/jmammal/gyae048

**Published:** 2024-05-07

**Authors:** Julie-Pier Viau, Daniel Sigouin, Martin-Hugues St-Laurent

**Affiliations:** Centre for Forest Research, Département de Biologie, Chimie et Géographie, Université du Québec à Rimouski, 300 allée des Ursulines, Rimouski, QC G5L 3A1, Canada; Parks Canada, Forillon National Park, 1501 boul. Forillon, Gaspé, QC G4X 6M1, Canada; Centre for Forest Research & Centre for Northern Studies, Département de Biologie, Chimie et Géographie, Université du Québec à Rimouski, 300 allée des Ursulines, Rimouski, QC G5L 3A1, Canada

**Keywords:** biological period, carnivore, GPS telemetry, habitat selection, limiting factor, *Martes americana*, mustelid, protected area

## Abstract

Old-growth forests harbor a large amount of complex structural features that result in a wide array of wildlife habitats. However, intensive forest management is gradually converting old-growth forest into younger, even-aged stands, reducing structural complexity and threatening the persistence of old-growth-dependent species. Maintaining elements of complex stand structure is critical to the conservation of old-growth forest specialists that use different habitat components at different periods of their annual cycle, and it requires a comprehensive understanding of seasonal variation in the habitat needs of these species. However, difficulties in observing free-ranging animals have sometimes limited our ability to assess such variations in habitat requirements, especially for small, elusive species. To address this, we used GPS telemetry collars to describe fine-scale habitat selection patterns of 6 male American Martens (*Martes americana*) during 2 contrasting periods of the year (snow-free, from mid-April to mid-November; snow-covered, from mid-November to mid-April), an objective formerly hard to achieve using conventional VHF telemetry. We used resource selection functions conducted at the fourth order of selection to compare habitat characteristics found at the sites used by martens (GPS locations, *n* = 100) to those found on an equal number of available sites (random points, *n* = 100) within each individual seasonal home range. We conducted vegetation surveys on these 200 sites to describe habitat and built candidate models representing different concurrent hypotheses. Our results showed that proxies of prey availability, predator avoidance, and thermal constraints were the primary factors influencing marten habitat selection during both periods, although their respective importance differed between periods. Martens selected sites with a high density of large-diameter snags (≥30·ha^−1^), high conifer canopy closure (≥53%), and a dense lateral cover (≥81%) during the snow-free period, but selected sites with a high volume of coarse woody debris (≥64 m^3^·ha^−1^) and high conifer canopy closure (≥48%) during the snow-covered period. Our results highlight the importance of contrasting seasonal changes in habitat selection patterns of small carnivores and may help maintain structural attributes in the landscape that are suitable for male American Martens.

## Teaser

The habitat selection of American Marten has rarely been studied using GPS telemetry; few studies have described how it is influenced by seasons. Recent advances in the miniaturization of GPS devices allowed us to model seasonal variation in fine-scale habitat selection.

Global biodiversity loss is one of the greatest concerns of the 21st century (Barnosky et al. 2011). Anthropogenic habitat loss is a major driver of the current biodiversity crisis, as it removes resources and conditions that are essential for the survival and reproduction of organisms (Pimm et al. 2014; Chase et al. 2020). Old-growth forests, which are among the most species-rich terrestrial ecosystems on Earth, are undergoing increasing alteration (Betts et al. 2022), resulting in habitat loss and fragmentation for several late-successional wildlife species (Wich et al. 2003; Noon and Blakesley 2006). Old-growth forests typically contain a variety of complex vertical and horizontal structures including large decaying tree trunks, multilayered canopies, and coarse woody debris, that allow individuals to meet their habitat requirements (Le Roux et al. 2014; Lindenmayer et al. 2014). However, these features are generally absent or poorly represented in young natural forests and in intensively managed, often even-aged, forests (Arnett et al. 2010; Bouget et al. 2014). Therefore, maintaining elements of complex stand structure that are required to sustain old-growth forest specialists has become an urgent task (Beese et al. 2019; Ettwein et al. 2020).

Among different approaches, habitat selection analyses are commonly used to identify critical habitats for wildlife (Holbrooke et al. 2017; Pop et al. 2018). Resource selection functions (RSFs; Manly et al. 2002) are a robust tool commonly used to contrast environmental predictor variables at locations used by an animal with those found randomly at available locations in the environment (Boyce et al. 2002; Johnson et al. 2006; Northrup et al. 2013). Habitat selection also operates at multiple spatial scales (Dussault et al. 2005; McGarigal et al. 2016)—within the geographic range of a species (coarse scale), individuals select a home range within which they restrict movements and choose specific resources to meet requirements associated with their foraging and antipredator behaviors (fine scale; sensu Johnson 1980). Habitat selection analyses conducted at a fine spatial scale are very useful for local conservation practices (Gilioli et al. 2018; Huon et al. 2021), including habitat restoration (Martelo et al. 2021), and are especially valuable for species associated with complex structural habitat characteristics (Banks and Skilleter 2007; McCann and Zollner 2014).

Resource selection is also known to reflect a species habitat requirements that vary seasonally, forcing analyses to be conducted at the appropriate temporal scales (Boyce et al. 2006; Mayor et al. 2009). This is of utmost importance for species inhabiting temperate ecosystems with predictable seasonal shifts in resource needs and availability (Ellington et al. 2020; Martin et al. 2021). For example, Cougars (*Puma concolor*) in Canada selected wetlands and avoided forests in summer, while the opposite relationship was observed in winter, probably because prey change habitat seasonally (Smereka et al. 2020). In line with this, Red Deer (*Cervus elaphus alxaicus*) in China selected closed-canopy habitats and avoided open areas in winter, but choose open areas and avoided closed-canopy covers in summer, likely due to seasonal changes in the relative importance of thermal stress and predation risk (Zhang et al. 2013). Fishers (*Martes pennanti*) in British Columbia were shown to select different types of resting structures depending on the local ambient temperature; coarse woody debris were more frequently used when ambient temperature was low, while branches and cavities were most often used when ambient temperature was high (Weir et al. 2005). Habitat selection studies that consider seasonal variation in resource needs can provide critical information that improve the effectiveness of habitat management practices (Apps et al. 2001; Uboni et al. 2015). However, difficulties in observing free-ranging animals have sometimes limited the ability to assess temporal patterns of resource selection (Recio et al. 2011).

Over the past few decades, telemetry has been a reliable research tool for studying the ecology of free-ranging animals (Cooke et al. 2004; Cagnacci et al. 2010). Very high frequency (VHF) transmitters have been widely used and allowed researchers to study the ecology of many wildlife species (Martin et al. 2009). However, collecting VHF data can be logistically challenging, reducing the ability to make inferences over the entire daily cycle (Hebblewhite et al. 2010) and potentially influencing the behavior of collared animals when approaching them prior to triangulation (White and Garrott 1990; Rodgers et al. 1996). In the early 1990s, the advent of Global Positioning System (GPS) devices greatly reduced the time-consuming methods of VHF telemetry and allowed for the collection of more accurate and more frequent locations across all daily phases with less influence on animal behavior (Cagnacci et al. 2010; Recio et al. 2011). Although GPS telemetry has been a major technological advancement, it has been implemented primarily on large animals due to the weight, size, and battery life limitations of collars. However, recent progress in the miniaturization of GPS tags allows them to be deployed on smaller animals (<1 kg), providing opportunities to improve knowledge of their habitat requirements (McMahon et al. 2017).

The American Marten (*M. americana*) is a small carnivore (500 to 1400 g; Buskirk and Ruggiero 1994) commonly found in most North American forests (Hall 1981). Reduction of the southeasternmost limits of the historical distribution range has been noted, with some marten populations extirpated largely because of unregulated commercial trapping and loss of mature forests (Laliberté and Ripple 2004). Martens are closely associated with complex forest structures that include dense canopy (Thompson 1994) and lateral cover for prey availability and predator avoidance (Godbout and Ouellet 2010), coarse woody debris for prey accessibility and thermoregulation (Payer and Harrison 2003), large trees to escape terrestrial predators (Chapin et al. 1997), and large snags for shelter and thermoregulation (Porter et al. 2005; Sanders et al. 2017). Although the fine-scale seasonal habitat selection patterns of American Marten have been studied previously (Thompson et al. 2012), most studies have used VHF telemetry, thus potentially limiting the precision of the patterns described. For now, the fine-scale habitat selection patterns of American Marten were shown to be associated with the fulfillment of food requirements and reduction of predation risk and thermal constraints (Thompson et al. 2012). During the snow-covered period, martens often rely on a limited food supply (Hales et al. 2008), which can favor the selection of sites offering greater foraging opportunities. Martens are often more vulnerable to predation during the snow-covered period because of their dark brown fur, which contrasts with the white snow cover (Bull and Heater 2000). Furthermore, martens may have higher thermal constraints during the snow-covered period due to their low-fat reserves and colder air temperatures (Buskirk et al. 1989; Wilbert et al. 2000). Thus, selecting sites offering lower predation risk and higher thermal cover is potentially a priority during the snow-covered period. During the snow-free period, marten diets generally become more diverse (Buskirk and Powell 1994)—feeding opportunities are therefore greater, which may reduce the intensity of selection among feeding sites. The selection of sites with lower predation risk and greater thermal cover may also be less important during the snow-free period.

In this study, we capitalize on recent advances in the miniaturization of GPS technology devices to model seasonal variation in fine-scale habitat selection patterns of male American Martens. We considered 2 distinct periods based on the presence of snow on the ground—i.e., the snow-free and the snow-covered periods—considering that the presence of snow can influence resource availability and accessibility (e.g., food, shelter), thermal constraints, and vulnerability to predators, with consequences on the habitat selection patterns of several wildlife species (e.g., Parker et al. 1984; Briand et al. 2009), including American Marten. Based on current knowledge of marten ecology, we hypothesized that fine-scale habitat selection patterns of martens are driven by prey availability and accessibility (hereafter referred to as prey availability) as well as predator avoidance during the snow-free period—but by prey availability, predation risk, and thermal constraints during the snow-covered period (Godbout and Ouellet 2010; Thompson et al. 2012). Thus, we predicted that martens would select for sites where they can find a dense coniferous canopy cover, dense lateral cover, and high volume of coarse woody debris (proxies for prey availability and predator avoidance) during the snow-free period, but would select for large trees, a high snag density, dense coniferous cover, dense lateral cover, and high volume of coarse woody debris (proxies for prey availability, predator avoidance, and thermal constraints) during the snow-covered period.

## Materials and methods

### Study area

**Fig. 1.**
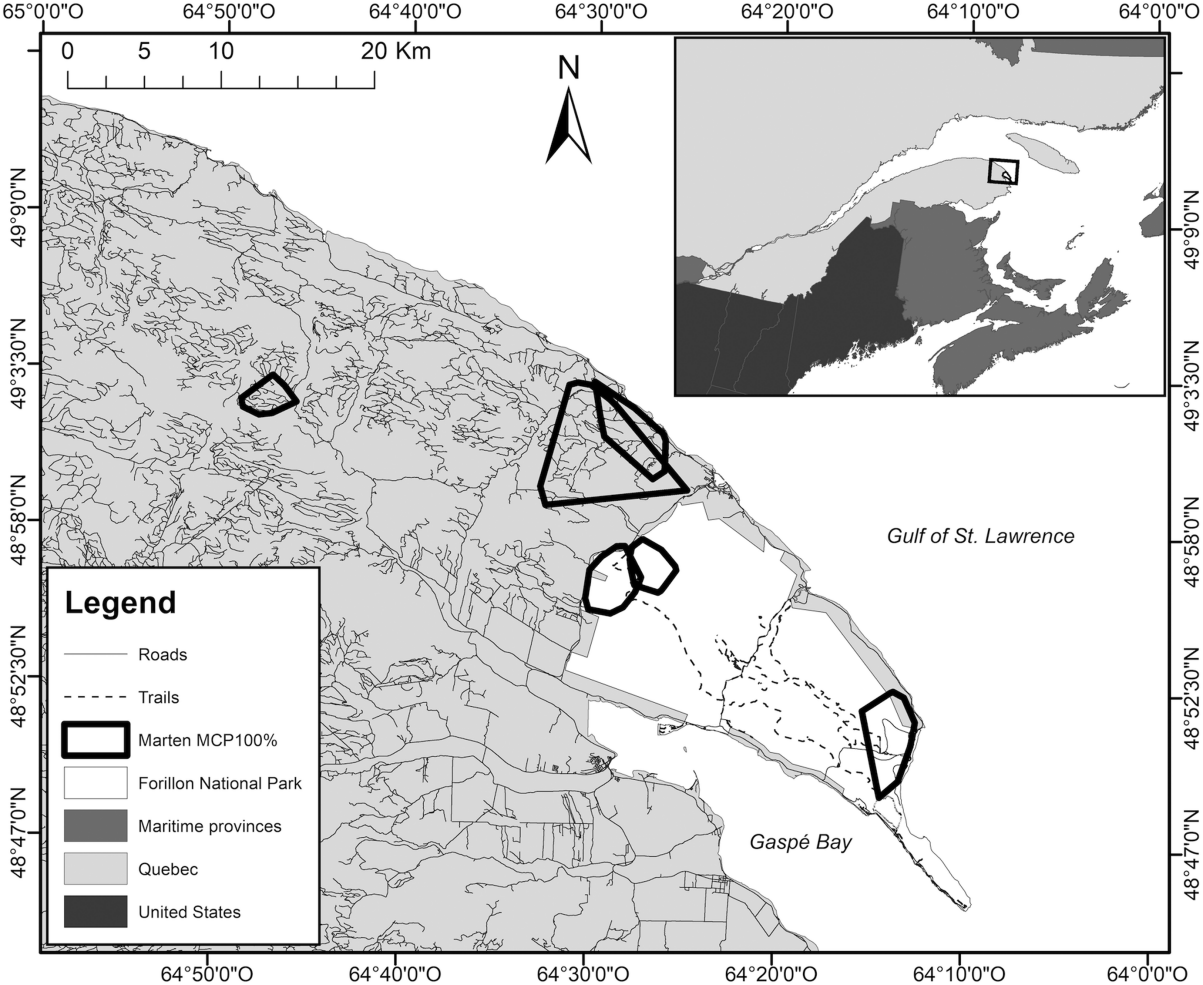
Map of our study area located in eastern Québec, Canada (top-right insert), which covers Forillon National Park (white polygon) and the surrounding public and private lands west of Highway 197 (i.e., the western boundary of the park). The map also shows the home range of 6 martens (polygons delineated with thick black lines), roads (thin, solid black lines), and hiking trails (dotted black lines).

Our study area encompasses Forillon National Park (244 km^2^) and its surrounding area, including parts of the public and private lands located west of Highway 197 (466 km^2^), in eastern Quebec, Canada (Fig. 1). The study area has mountainous and hilly terrain, with elevations ranging from sea level up to 736 m. It also has a humid continental climate, with a mean ambient temperature of 17 °C in July and −10 °C in January (Environment and Climate Change Canada 2022). Annual precipitation averages 1,000 mm, with annual snowfall reaching ~2,600 mm (Environment and Climate Change Canada 2022). The Gaspé Peninsula is generally covered by snow from mid-November to late April. The study area occurs in the Balsam Fir (*Abies balsamea*)–Yellow Birch (*Betula alleghaniensis*) bioclimatic domain, where Balsam Fir, Eastern White Cedar (*Thuja occidentalis*), White Spruce (*Picea glauca*), Yellow Birch, White Birch (*Betula papyrifera*), and maples (*Acer* spp.) are the dominant tree species (Pinna et al. 2009). While no timber harvesting has occurred in the national park territory since 1970, the outlying area is still managed, creating a landscape dominated by young forests interspersed with old-growth coniferous stands. Forillon National Park is predominantly composed of mixed and second-growth hardwood stands.

### Capture, handling, and collaring protocols

We captured martens using certified live traps (Tomahawk model 202, Hazelhurst, Wisconsin, and Havahart model 1078, Lancaster, Pennsylvania) deployed between September and December 2020. We placed traps in wooden boxes covered with fir branches and fixed them on raised tree trunks to protect captured animals from harsh weather conditions and potential predators. We baited traps with Beaver (*Castor canadensis*) meat and added a commercial scent lure (XLDC, extra long-distance calls, Forget Lures Inc.) on a tree outside the trap. The bait was replaced as needed, and the lure was refreshed every week. We visited all capture sites daily before noon to minimize the time that captured animals spent in the traps. We anesthetized captured martens with an intramuscular injection of BAM (combination of butorphanol, azaperone, and medetomidine; Wildlife Pharmaceuticals, Inc., Windsor, Colorado), and fitted them with Litetrack 20 GPS collars (Lotek Engineering Inc., Newmarket, Ontario, Canada) if they weighed ≥650 g to make sure that the collar represented <3% of their body weight. GPS collars were programmed to attempt location fixes every 6 h for nearly 10 months. We determined the sex of each marten and estimated their age (i.e., juvenile and adult) based on teeth condition (i.e., wear and coloration). We also took various morphological measurements, including weight, neck circumference, hind foot length, tail length, and total length. Once the measurements were completed, we reversed the effects of the anesthesia with an intramuscular injection of both atipamezole and naltrexone (Wildlife Pharmaceuticals, Inc., Windsor, Colorado) and released martens at their point of capture once they were fully recovered. Our American Marten trapping and manipulation protocols were approved by the Animal Care Committee of the Université du Québec à Rimouski (2020 to 2022, certificate #CPA-81-20-221) and adhere to the guidelines of the American Society of Mammalogists (Sikes 2016).

### Collection and processing of GPS location data

We collected GPS data from collared martens for ~10 months (i.e., the autonomy of the collar battery), ranging between September 2020 and October 2021 depending on the date of capture. We attempted to locate martens using a portable 3-element Yagi antenna connected to a VHF receiver (PinPoint VHF Commander, Lotek Engineering Inc., Newmarket, Ontario, Canada). Once an animal was located, we approached the signal until the radio receiver was able to download GPS locations (~100 m). Nevertheless, the majority of the data were recovered through live-trapped recaptures or trapper-harvested individuals. Recaptures were conducted from September to December 2021, according to the capture protocol described above and during the period of validity of our Animal Care certificate. We then discarded GPS outliers, i.e., locations with high dilution of precision (2D and 3D fixes with DOP > 10; D’Eon and Delparte 2005; Cargnelutti et al. 2007) as well as obvious outliers (e.g., locations falling far from our study area, following Coulon et al. 2008). Location data were visualized and processed using R 4.1.1 (R Core Team 2021).

### Accuracy of GPS collars

We assessed the accuracy of our GPS collars in 2 contrasted environments, i.e., open and closed-canopy forests. A GPS collar programmed to take a location fix every 15 min was placed above a ground marker (i.e., reference point) in each of the 2 environments for approximately 48 h. The location of the ground marker was recorded with a GNSS smart antenna (Leica Viva GS14, Leica Geosystems, Heerbrugg, Switzerland). Following our protocol for collecting and processing GPS location data, we discarded locations with 2D and 3D fixes with DOP > 10. To evaluate the accuracy of our GPS collars, we calculated the average distance between all locations and the reference point for both the open and the closed-canopy forests.

### Fine-scale habitat characterization

In order to conduct our habitat selection analysis at the fourth scale of selection (sensu Johnson 1980), we characterized forest structure on a subset of marten locations collected between September 2020 and the end of June 2021, i.e., the start of the summer fieldwork season. To reflect seasonal variation in resource selection, we partitioned GPS locations into 2 periods: the “snow-covered” period (from 15 November to 11 April) and the “snow-free” period (from 12 April to 14 November). We used the snow accumulation data for the year of our study from the Gaspé weather station (adjacent to the park; Environment and Climate Change Canada 2022) to determine the beginning and end dates of each period. We used a stratified sampling approach and sampled, for each period, 50 marten locations for a total 100 “used” sites. We distributed the sampling equally among all individuals to avoid 1 marten having a disproportionate influence. We made sure to avoid selecting successive GPS locations to avoid temporal pseudoreplication and to cover most of the period duration; on average, and without considering the marten ID, GPS locations were spaced by 1.4 days during the snow-free period and by 2.2 days during the snow-covered period. As we were not interested in contrasting patterns of habitat selection between day and night, we did not select GPS locations to make sure to cover evenly each phase of the day. Additionally, we maintained a minimum distance of 100 m between different sampling sites to ensure sample independence (following Bowman and Robitaille 1997). Home ranges were delineated with 100% minimum convex polygons (Mohr 1947) to emphasize the contrast between used and available locations (Gillies et al. 2006). Random locations were distributed within each individual home range in the same proportion of the GPS locations we used to represent sites available to an individual (Leclerc et al. 2012), for a total of 50 random points per period (100 in total). We used ArcGIS 10.7.1 and R 4.1.1 (R Core Team 2021) software to perform spatial analyses and the selection of sampling sites (*n* = 200).

The vegetation surveys were carried out from late June to August 2021. When visiting each sampling site, we measured 8 habitat characteristics, already known to be important for marten, to describe the structural complexity of forest stands in an 11.28-m-radius (400 m^2^) circular plot. We measured percent canopy closure using a spherical densitometer centered on each sampling site and averaging values taken in each cardinal direction (Lemmon 1956). We considered all features likely to provide overhead cover including leaves, branches, and tree trunks, and assessed canopy closure separately for coniferous and deciduous tree species. We counted, identified to the species level, and measured the diameter at breast height (i.e., 1.3 m above ground level, hereafter DBH) of all trees with a DBH ≥ 9.1 cm (Potvin et al. 1999). We also counted and measured the DBH of all snags ≥ 9.1 cm. We measured the percentage of lateral cover, i.e., the visual obstruction caused by all horizontal features, at 15 m from the center of the plot and in each cardinal direction using a 2-m-high vegetation profile board (Nudds 1977). Measurements were taken in a kneeling position to approximate the height of the head of a marten. We estimated lateral cover between 0 and 2 m high for the snow-free period and between 1 and 2 m high for the snow-covered period because snow cover typically reaches at least 1 m high in our study area (Environment and Climate Change Canada 2022). Finally, we counted all items of coarse woody debris with a diameter ≥ 9.1 cm intercepting a 15-m transect in a cardinal direction selected randomly using a 4-sided die (adapted from Bertrand and Potvin 2003).

### Statistical analyses

We used resource selection functions (hereafter RSFs; Manly et al. 2002) to assess the habitat selection of American Martens at Johnson’s fourth order of selection (sensu Johnson 1980). For each period, we used conditional logistic regression (Breslow et al. 1978) to compare habitat characteristics found at the sites used by martens (i.e., GPS locations) with those found at the sites available in individual home ranges (i.e., random locations). To account for interindividual variation in resource selection, we used individuals as a conditional stratum. Before conducting our analyses, we checked for multicollinearity between all of our independent variables using the variation inflation factor (Graham 2003). For each period, we built different candidate models representing plausible biological hypotheses and ranked them with the Akaike Information Criterion corrected for small sample size (AIC_c_; Burnham and Anderson 2003). Then, we evaluated the robustness of the top-ranked models using *k*-fold cross-validation and the Spearman rank coefficient (Boyce et al. 2002). Our candidate models represent groups of environmental variables known to influence the fine-scale habitat selection of martens: prey availability (Koehler and Hornocker 1977; Godbout and Ouellet 2010), predator avoidance (Bull et al. 2005), and thermal constraints (Buskirk et al. 1989; Corn and Raphael 1992; see Table 1 for model description). We used conifer canopy closure (%), lateral cover (%), and the volume of coarse woody debris (m^3^·ha^−1^) as proxies to describe prey availability, as some marten prey species (e.g., snowshoe hare and voles) are positively associated with these environmental variables (Beaudoin et al. 2004; Etcheverry et al. 2005). Tree diameter (cm), conifer canopy closure, and lateral cover were used as proxies to describe predator avoidance since these variables are known to provide escape cover from terrestrial and avian predators (Godbout and Ouellet 2010). The density (stems·ha^−1^) of large-diameter snags (DBH > 20 cm) and the volume of coarse woody debris were used as proxy variables to describe thermal constraints, as these structures are known to provide protection against harsh weather conditions (Porter et al. 2005). For both annual periods, the first 3 models allowed us to evaluate the respective influence of prey availability, predation risk, and thermal constraints on marten habitat selection patterns, while the fourth model allowed us to assess whether habitat selection patterns represent trade-offs between these limiting factors (Table 1). We used R 4.1.1 R Core Team (2021) to perform all statistical analyses.

**Table 1.** Candidate models used to describe American Marten habitat selection during 2 contrasting periods in Forillon National Park and its periphery (Québec, Canada) between 2020 and 2021. Candidate models are presented with their number of parameters (*K*), log-likelihood (LL), and difference in AIC_c_ (ΔAIC_c_). The most parsimonious model (for each season) is shown in bold.

Hypotheses	Variables	*K*	LL	ΔAIC_c_
Snow-free period				
1—Prey availability	Coniferous canopy closure + Coarse woody debris + Lateral cover (0 to 2 m above ground level)	3	−35.17	5.16
2—Predator avoidance	Tree diameter + Coniferous canopy closure + Lateral cover (0 to 2 m above ground level)	3	−35.20	5.22
3—Thermoregulation	Snag density (DBH > 20 cm) + Coarse woody debris	2	−55.89	44.59
**4—Complete**	**Model 1 + Model 2 + Model 3**	**5**	**−30.59**	**0.00**
Snow-covered period				
1—Prey availability	Coniferous canopy closure + Coarse woody debris + Lateral cover (1 to 2 m above ground level)	3	−41.88	0.18
2—Predator avoidance	Tree diameter + Coniferous canopy closure + Lateral cover (1 to 2 m above ground level)	3	−52.97	22.36
3—Thermoregulation	Snag density (DBH > 20 cm) + Coarse woody debris	2	−46.37	7.16
**4—Complete**	**Model 1 + Model 2 + Model 3**	**5**	−**39.79**	**0.00**

## Results

### Animal captures

From September to December 2020, we captured 16 individual martens (14 males and 2 females) over 1935 trap-nights and fit 10 males (≥650 g) with GPS collars, as females were too light to be collared. We relocated or recaptured 6 males before the start of the forest inventory season, i.e., in late June 2021, and downloaded a total of 1,194 GPS locations. A total of 725 GPS locations were recorded during the 4½ months of the snow-free period while 469 were recorded during the 5 months of the snow-covered period. After removing location errors (i.e., 2D and 3D fixes with DOP > 10), we had a total of 1,038 GPS locations of which 640 and 398 were recorded during the snow-free and the snow-covered periods, respectively. The average successful fix rates of our GPS collars were 53% and 40% during the snow-free and the snow-covered periods, respectively. GPS location accuracy (for 2D and 3D fixes with DOP < 10) was 14 m in open forest habitat and 35 m in closed forest habitat.

### Fine-scale resource selection during the snow-free period

**Fig. 2.**
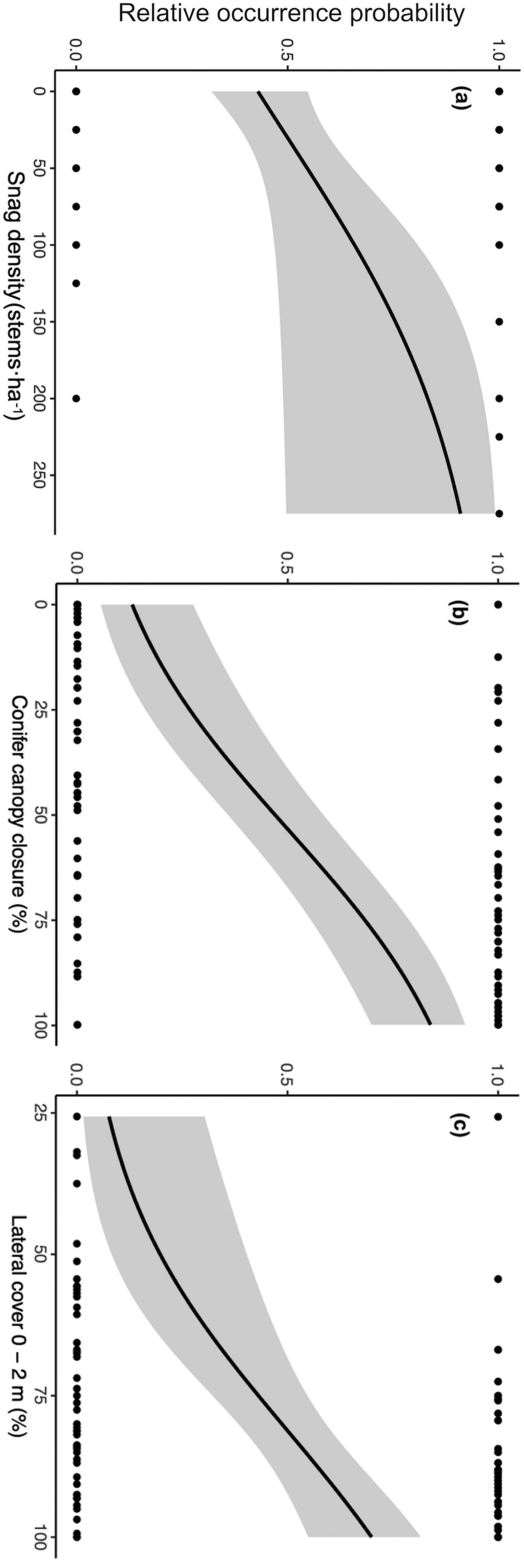
Relative occurrence probability of American Marten predicted according to: (a) snag density (DBH > 20 cm; stems·ha^−1^); (b) conifer canopy closure (%); and (c) lateral cover (%) during the snow-free period in Forillon National Park and its periphery (Québec, Canada) between 2020 and 2021.

The mean values of several habitat covariates differed between used (GPS locations) and random sites (Supplementary Data SD1). Habitat selection patterns of martens were best explained by Model 4 (i.e., the complete model), which included variables referring to prey availability, predator avoidance, and thermal constraints (Table 1). The probability of marten occurrence was higher at sites containing a high percentage of both conifer canopy closure (≥53%) and lateral cover in the strata 0 to 2 m above ground level (≥81%; Table 2; Fig. 2; Supplementary Date SD2). In addition, compared to random sites, martens selected sites where the density of large-diameter snags (DBH > 20 cm) was greater than 30 snags·ha^−1^ (Table 2; Supplementary Data SD2). Despite our small sample size (*n* = 6), the top-ranked model had a relatively good fit, with a Spearman *r*_s_ of 0.79 ± 0.12.

**Table 2.** Coefficients (β) and 95% confidence interval (95% CI [lower bound: upper bound]) of the covariates used in the most parsimonious model to describe American Marten habitat selection during 2 annual periods in Forillon National Park and its periphery (Québec, Canada) between 2020 and 2021. Significant variables are shown in bold.

Model	Variables	Snow-free period		Snow-covered period	
		β	95% CI	β	95% CI
4—Complete	Tree diameter	−0.091	[−0.274: 0.093]	−0.092	[−0.215: 0.031]
	Snag density (DBH > 20 cm)	**0.698**	**[0.207: 1.189]**	0.303	[−0.025: 0.632]
	Coarse woody debris	−0.002	[−0.012: 0.007]	**0.016**	**[0.008: 0.024]**
	Coniferous canopy closure	**0.050**	**[0.028: 0.072]**	**0.021**	**[0.005: 0.038]**
	Lateral cover (0 to 2 m above ground level)	**0.100**	**[0.049: 0.150]**	—	—
	Lateral cover (1 to 2 m above ground level)	—	—	0.027	[−0.001: 0.056]

### Fine-scale resource selection during the snow-covered period

**Fig. 3.**
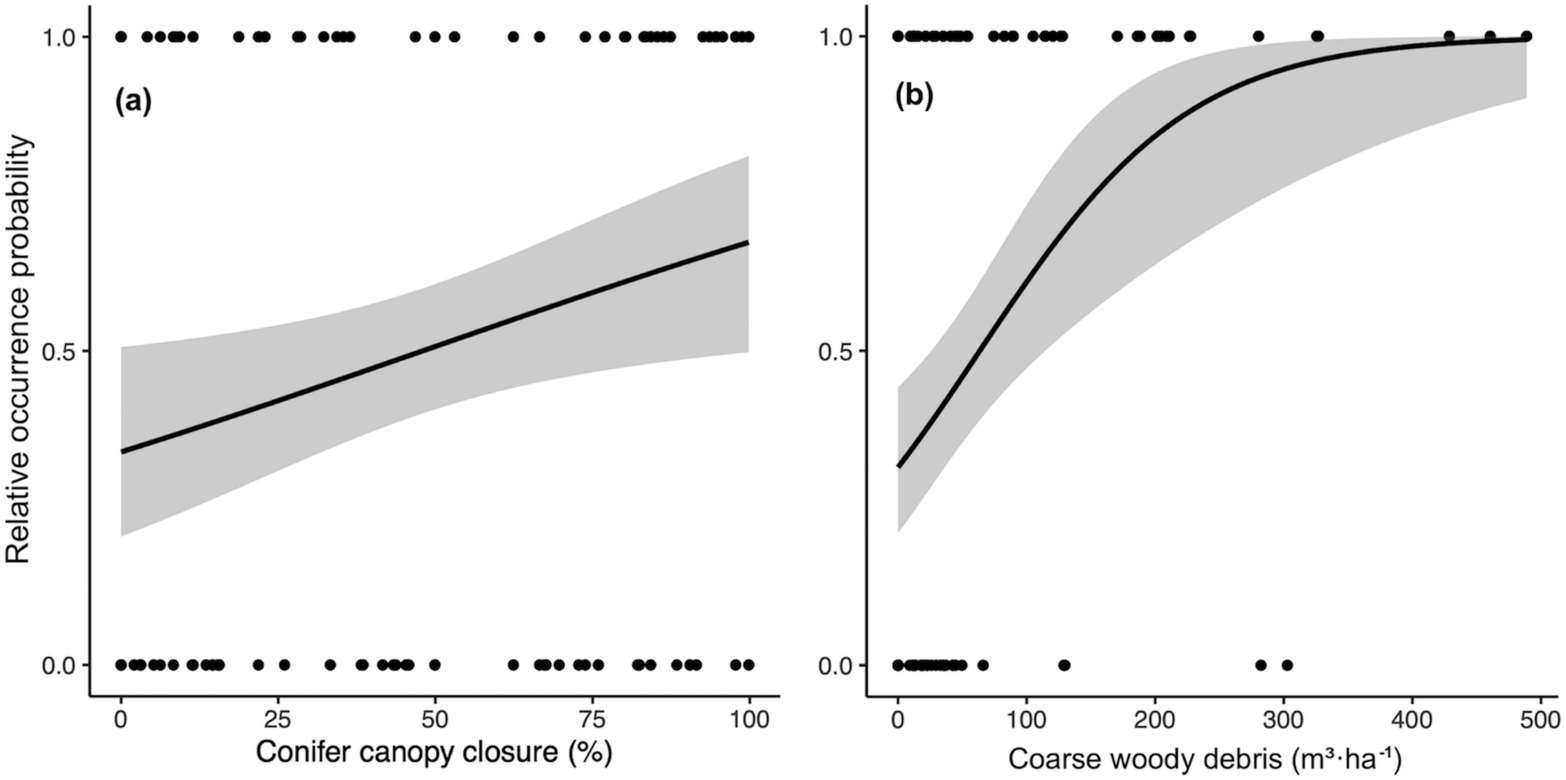
Relative occurrence probability of American Marten predicted according to: (a) coniferous canopy closure (%); and (b) volume of coarse woody debris (m^3^·ha^−1^) during the snow-covered period in Forillon National Park and its periphery (Québec, Canada) between 2020 and 2021.

During this period, Model 4 (i.e., the complete model) was also the most parsimonious model (Table 1). However, Model 1, which included only variables relating to prey availability, also provided comparable support to our data (ΔAIC_c_ = 0.18; Table 2). Nevertheless, we only discuss the results of Model 4, as it contains all of the habitat covariates used in Model 1 (but see Supplementary Data SD3 for the coefficients and 95% CI of Model 1). We noticed a great contrast between mean values measured at used (GPS locations) and random sites for several covariates describing structural complexity of forest stands (Supplementary Data SD1). The probability of marten occurrence was positively correlated with the percentage of conifer canopy closure and the volume of coarse woody debris (Table 2). Martens were using sites where the percentage of conifer canopy closure was greater than 48% and where the volume of coarse woody debris was greater than 64 m^3^·ha^−1^ (Fig. 3; Supplementary Data SD2). Furthermore, martens tended to select sites with a greater density of large-diameter snags (DBH > 20 cm) and a higher percentage of lateral cover between 1 and 2 m above ground level (Table 2; Supplementary Data SD1). However, as these variables were only marginally significant, these results should be interpreted with caution. The result of the cross-validation was lower than that of the snow-free model, with a Spearman *r*_s_ of 0.62 ± 0.14, but was still satisfying considering our limited sample size.

## Discussion

Our study aimed to describe fine-scale habitat selection patterns of 6 male American Martens during 2 contrasting periods of the year using GPS collars. We showed that annual periods, which are typically not considered when studying the habitat selection of martens (Tweedy et al. 2019), play an important role in explaining fine-scale habitat selection. Our results indicate that proxy variables of prey availability, predation risk, and thermal constraints influenced marten habitat selection, as the most parsimonious model retained was the complete model for both annual periods. This suggests that all variables we tested are relevant to marten biology, although the effect of the proxies representing thermal constraints had only an effect on the fine-scale habitat selection patterns of martens during the snow-covered period.

### Fine-scale resource selection during the snow-free period

In accordance with our predictions, martens were more likely to use sites with characteristics that favor prey availability and predator avoidance. Firstly, the probability of marten occurrence was higher when the density of conifer canopy closure and lateral cover 0 to 2 m above ground level were high, with respective thresholds of 53% and 81%. Although we did not directly measure prey availability, studies conducted close to our study area showed that conifer canopy closure and lateral cover favored 2 major prey species of marten: the Snowshoe Hare (*Lepus americanus*; Beaudoin et al. 2004) and the Red-backed Vole (*Myodes gapperi*; Etcheverry et al. 2005), which are both present in our study area. Lateral cover is known to reduce the hunting efficiency of predators by providing physical refugia for prey (White and Berger 2001) or by impeding the movements of predators across the landscape (Whittington et al. 2005). However, lateral cover could also increase hunting efficiency if it reduces the escape ability of prey (Lima 1992) or makes predators less visible to prey (Moreno et al. 1996). In line with this, Andruwski et al. (2008) showed that American Martens captured more prey in structurally complex versus simpler stands—even though prey density was similar in both stand types—suggesting that complex structural features may enhance, rather than diminish, marten hunting efficiency. On the other hand, lateral cover is known to impede movements of Coyotes (*Canis latrans*; Richer et al. 2002; Thibault and Ouellet 2005) and Lynx (*Lynx canadensis*; Murray et al. 1995; Fuller et al. 2004), which are 2 potential predators of martens in our study area. Therefore, we interpret this association with dense lateral cover as a way for martens to increase prey encounters in areas where prey availability is higher while segregating from terrestrial predators.

Secondly, collared martens selected sites containing a high density of large-diameter snags (higher than 30 snags per ha), suggesting that thermal constraints also influence habitat selection patterns during the snow-free period. Snags, especially larger ones, often contain cavities that provide suitable resting sites used to avoid harsh weather conditions (Buskirk 1984; Zalewski 1997). Typically, structures used for resting vary throughout the year, depending on their availability and environmental conditions (Bull et al. 2005). For example, Bull and Heater (2000) showed that American Martens used snag cavities year-round, especially in fall and spring when ambient temperatures are cold and rainfall is frequent (Bull and Heater 2000). Under such weather conditions, martens need dry and insulated resting structures, which can be provided by snag cavities (Martin and Barrett 1991).

### Fine-scale resource selection during the snow-covered period

We found that martens selected sites containing a high volume of coarse woody debris (greater than 64 m^3^·ha^−1^), a structural habitat feature potentially linked to prey availability and thermoregulation. A higher volume of coarse woody debris could intercept snowfall, facilitating access to the subnivium, i.e., the microhabitat at the snow–ground interface (Harmon et al. 1986). Subnivean sites are recognized as suitable marten microhabitats, as they provide prey and thermal cover (Pulliainen 1981; Buskirk 1984). In fact, several prey species of martens, such as voles, mice (*Peromyscus* spp.), and shrews (*Sorex* spp.), spend most of the winter under the snowpack (Pruitt 1984). In addition, subnivean sites are enclosed, insulated microenvironments that provide thermally efficient resting sites during periods of cold weather (Martin and Barrett 1991; Taylor & Buskirk, 2006; Sanders et al. 2017).

We also showed that sites with a high conifer canopy closure had a greater probability of use by martens, with a threshold ≥64%, providing additional support to the hypothesis that prey availability and predator avoidance are important factors influencing marten habitat selection during the snow-covered period. In addition to increased prey availability (Godbout and Ouellet 2010), a dense conifer canopy closure could provide protective cover from avian predators, such as Golden Eagles (*Aquila chrysaetos*) and Great-horned Owls (*Bubo virginianus*; Hargis and McCullough 1984; Thompson 1994). This variable could be even more important during the snow-covered period, as martens are more visible on the snow surface and, therefore, more vulnerable to predation (Buskirk and Powell 1994; Bull and Heater 2000).

Finally, our results indicated that martens tend to select sites where the density of large-diameter snags and lateral cover 1 to 2 m above ground level was high. We suspect that the marginally significant selection toward these 2 variables results from our small sample size, which potentially reduces the statistical power of our analyses and our ability to detect weaker effects (Hebblewhite et al. 2010). However, as these variables were only marginally significant, these results should be interpreted with caution. Large snags can also provide thermally efficient resting sites during the snow-covered period, especially in early and late winter when snow accumulations are generally not sufficient to form suitable subnivean resting sites (Bull and Heater 2000). Moreover, lateral cover can be an important habitat feature for martens during the snow-covered period, as it provides protective cover from terrestrial predators (Chapin et al. 1998) and is a good proxy of prey availability (Litvaitis et al. 1985; Beaudoin et al. 2004). Lateral cover may be of particular importance during the snow-covered period because of the greater visibility of martens in the snow cover (Godbout and Ouellet 2010).

### Limitations

Our results are based on a small sample, i.e., 6 male martens, which potentially limits the generalization of our results to the entire population of martens occupying the Gaspé Peninsula (Lindberg and Walker 2007; Hebblewhite et al. 2010). Indeed, our small sample size may have biased the estimation of some regression coefficients, widened their confidence intervals and thus potentially limited our ability to make robust inferences at the population level (Leban et al. 2001). However, considering that none of our candidate models was overparametrized, we consider that our results are nevertheless reliable. In addition, considering that female martens may have different requirements compared to males (Hales et al. 2008; Simons-Legaard et al. 2022), it is unlikely that our results can be inferred to them. Moreover, although our GPS collars collected locations over a long period (~10 months), little data were collected during the 2 warmest months of the snow-free period (i.e., July and August), so sites used by martens during these months were rare in our forest inventories, as our sampling design was based on locations downloaded by the end of June; this may have slightly biased our inferences for this period. Finally, we did not include the day phase in our statistical models due to sample size constraints, although the GPS relocations visited were distributed as evenly as possible between the 4 daily periods available (3 AM, 9 AM, 3 PM, 9 PM) and between the collared individuals. We still consider that using GPS collars rather than VHF transmitters (as in most previous studies, e.g., Bowman and Robitaille 1997; Godbout and Ouellet 2010; Wiebe et al. 2014) was an interesting contribution despite the lower accuracy noted in the closed-canopy stands and the quite disappointing success rate (47%) of the small collars we used. In the absence of GPS collars that can provide a higher fix rate and more accurate relocations under canopy cover, we consider that we went a step further with this technology as our collars collected ~1,200 GPS locations distributed over 10 months and the entire daily cycle (i.e., day and night), which allowed us to establish more precise and fine-scale associations between martens and habitat characteristics (Cagnacci et al. 2010) and discern variations in habitat selection patterns in response to seasonal changes in environmental conditions.

Our results reinforce the consensus that the American Marten is a species associated with structurally complex forests (e.g., Thompson 1994; Chapin et al. 1997; Payer and Harrison 2003; Porter et al. 2005; Andruwski et al. 2008; Godbout and Ouellet 2010; Sanders et al. 2017). In addition, they add further support to previous studies that reported that American Martens attempt to maximize prey intake and avoid predation and heat stress during the snow-covered period by selecting sites with closed-canopy cover and coarse woody debris (Bowman and Robitaille 1997; Wiebe et al. 2014). However, we showed that martens in our study area are dealing with the same limiting factors during the snow-free period, while selecting for different features, such as closed canopies, dense lateral cover, and large-diameter snags. Therefore, our study demonstrates that assessing habitat selection over multiple periods can provide important ecological insights that are not necessarily provided in studies using a single biological period. Furthermore, considering that some variables measured in this study (e.g., lateral cover, coarse woody debris, snags) are currently unavailable in standard forest inventories, we recommend including them in order to maintain structural attributes favorable to martens. Finally, by identifying the habitat characteristics that best explain marten use in forest stands during 2 distinct periods and providing threshold values of different covariates above which martens are commonly found, our results will help guide land-use and forest managers to maintain suitable structural attributes for American Marten in areas devoted to resource extraction (e.g., timber harvesting), but also in nearby protected areas including the Forillon National Park, where we conducted our study.

## Supplementary data

Supplementary data are available at *Journal of Mammalogy* online.

**Supplementary Data SD1.**—Mean values (±SD) of the variables used to describe marten habitat selection as measured at used and available sites during the snow-free and snow-covered periods in Forillon National Park and its periphery (Québec, Canada).

**Supplementary Data SD2.**—Threshold values at which the relative probability of occurrence of American Marten passes ≥ 0.5, i.e., the probability that a site represents a GPS location of a marten instead of a random point, for each habitat variables that had a significant effect on the habitat selection patterns of male American Martens during the snow-free and snow-covered periods in Forillon National Park and its periphery (Québec, Canada).

**Supplementary Data SD3.**—Coefficients (β) and 95% confidence interval (95% CI [lower bound: upper bound]) of the covariates used in the second best model to describe marten habitat selection during 2 annual periods in Forillon National Park and its periphery (Québec, Canada) between 2020 and 2021. Significant variables are shown in bold.

gyae048_suppl_Supplementary_Data_1

gyae048_suppl_Supplementary_Data_2

gyae048_suppl_Supplementary_Data_3

## Data Availability

Our GPS telemetry data are available upon request.
