# Supplementary material for: Seasonal shifts in the habitat selection patterns of male American Marten (Martes americana) at a fine spatial scale
Source: J Mammal. 2024 May 7;105(4):740–51. doi: 10.1093/jmammal/gyae048 (PMC11285161; doi:10.1093/jmammal/gyae048)
Supplement: gyae048_suppl_Supplementary_Data_1 [file gyae048_suppl_supplementary_data_1.docx]

**Supplementary Data SD1**. Mean values (± SD) of the variables used to describe marten habitat selection as measured at used and available sites during the snow-free and snow-covered periods in Forillon National Park and its periphery (Québec, Canada).

| Variables | Snow-free period | |  | Snow-covered period | |
| --- | --- | --- | --- | --- | --- |
|  | Used | Available |  | Used | Available |
|  | Mean (± *SD*) | Mean (± *SD*) |  | Mean (± *SD*) | Mean (± *SD*) |
| Tree diameter (cm) | 17.5 ± 3.58 | 16.5 ± 4.24 |  | 18.9 ± 5.08 | 18.4 ± 5.37 |
| Snag density (stems⋅ha^-1^) | 43.5 ± 61.2 | 21.0 ± 40.8 |  | 49.5 ± 53.6 | 28.5 ± 38.1 |
| Coarse woody debris (m^3^⋅ha^-1^) | 57.2 ± 90.3 | 32.9 ± 67.7 |  | 123.0 ± 125.0 | 32.1 ± 62.6 |
| Coniferous canopy closure (%) | 70.4 ± 28.2 | 34.8 ± 31.4 |  | 56.2 ± 35.6 | 39.7 ± 32.9 |
| Lateral cover (0 – 2 m) (%) | 86.2 ± 13.7 | 74.4 ± 19.4 |  |  |  |
| Lateral cover (1 – 2 m) (%) |  |  |  | 73.8 ± 24.3 | 62.4 ± 26.5 |
