# Supplementary material for: Seasonal shifts in the habitat selection patterns of male American Marten (Martes americana) at a fine spatial scale
Source: J Mammal. 2024 May 7;105(4):740–51. doi: 10.1093/jmammal/gyae048 (PMC11285161; doi:10.1093/jmammal/gyae048)
Supplement: gyae048_suppl_Supplementary_Data_2 [file gyae048_suppl_supplementary_data_2.docx]

**Supplementary Data SD2**. Threshold values at which the relative probability of occurrence of American Marten passes ≥ 0.5, i.e., the probability that a site represents a GPS location of a marten instead of a random point, for each habitat variables that had a significant effect on the habitat selection patterns of male American martens during the snow-free and snow-covered periods in Forillon National Park and its periphery (Québec, Canada).

| Variables | Threshold values | |
| --- | --- | --- |
|  | Snow-free period | Snow-covered period |
| Snag density (stems⋅ha^-1^) | 30 | - |
| Conifer canopy closure (%) | 53 | 48 |
| Lateral cover (0 – 2 m) (%) | 81 | - |
| Coarse woody debris (m^3^⋅ha^-1^) | - | 64 |
