# Supplementary material for: Seasonal shifts in the habitat selection patterns of male American Marten (Martes americana) at a fine spatial scale
Source: J Mammal. 2024 May 7;105(4):740–51. doi: 10.1093/jmammal/gyae048 (PMC11285161; doi:10.1093/jmammal/gyae048)
Supplement: gyae048_suppl_Supplementary_Data_3 [file gyae048_suppl_supplementary_data_3.docx]

**Supplementary Data SD3.** Coefficients (*β*) and 95% confidence interval (95% *CI* [lower bound: upper bound]) of the covariates used in the 2^nd^ best model to describe marten habitat selection during two annual periods in Forillon National Park and its periphery (Québec, Canada) between 2020 and 2021. Significant variables are shown in bold.

| Model | Variables | Snow-free period | |  | Snow-covered period | |
| --- | --- | --- | --- | --- | --- | --- |
|  |  | *β* | 95% *CI* |  | *β* | 95% *CI* |
| 1 – Prey availability | Coniferous canopy closure | **0.043** | **[0.025 : 0.061]** |  | **0.018** | **[0.002 : 0.033]** |
|  | Coarse woody debris | 0.001 | [-0.007 : 0.009] |  | **0.015** | **[0.007 : 0.023]** |
|  | Lateral cover (0 – 2 m above ground level) | **0.087** | **[0.038 : 0.136]** |  | - | - |
|  | Lateral cover (1 – 2 m above ground level) | - | - |  | **0.032** | **[0.004 : 0.060]** |
